# Hypophosphatemic rickets due to Dent's disease: A case report and review of literature

**DOI:** 10.4103/0971-4065.59340

**Published:** 2009-10

**Authors:** R. A. Annigeri, R. Rajagopalan

**Affiliations:** Department of Nephrology, Apollo Hospitals, Chennai, India; 1 Department of Pediatrics, Apollo Hospitals, Chennai, India

**Keywords:** Dent's disease, nephrocalcinosis, rickets

## Abstract

We report a case of rickets due to Dent's disease in a two-year-old boy. He was treated with sodium phosphate, calcitriol and potassium citrate supplements, following which there was a remarkable improvement in mobility, growth and bony deformities. The hypercalciuria associated with Dent's disease was effectively corrected using hydrochlorothiazide.

## Introduction

Dent and Friedman first described Dent's disease in 1964, in two unrelated boys with rickets. Dent's disease is a rare X-linked recessive inherited proximal renal tubular disorder characterized by low molecular weight proteinuria (LMWP), hypercalciuria, nephrocalcinosis/nephrolithiasis, and progressive renal failure.[1–3] We report rickets due to Dent's disease in a two-year-old boy and review the literature on Dent's disease.

## Case Report

A two-year-old male child presented with an inability to walk since the age of one year. He was born to nonconsanguineous parents, the birth weight was 2.8 kg and he had normal early physical and mental milestones. He was able to stand and walk at 10 months, but within next few weeks he was unable to walk and stand even with support. A frontal bossing, potbelly appeared and there was a failure to gain height. He received calcitriol 0.25 μg on alternate days and elemental calcium 1 g /day, but showed no improvement.

**Figure 1:**
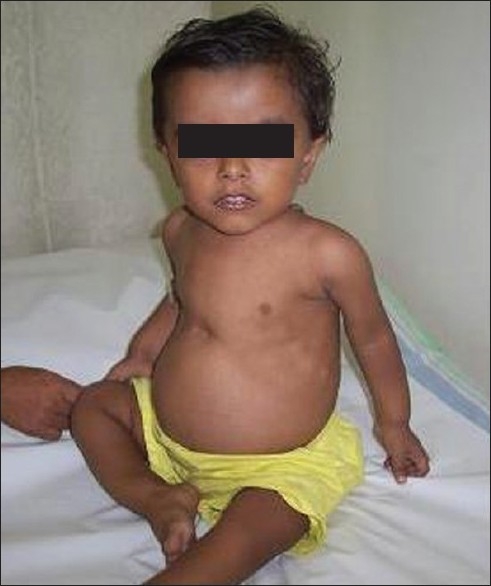
Child at presentation, showing bony deformities of rickets

On examination, he was irritable and blood pressure was 100/65 mmHg. He had no edema or pallor and anterior frontanelle was still open. He had classical features of rickets [Figure 1] such as frontal bossing, widened epiphysis at wrist, rickety rosary, potbelly and bowlegs. Systemic examination was unremarkable. Anthropometry showed height 73 cm, (5^th^ percentile), weight 9 kg (5^th^ percentle), head circumference 48 cm (between 75 and 90^th^ percentile).

**Figure 2:**
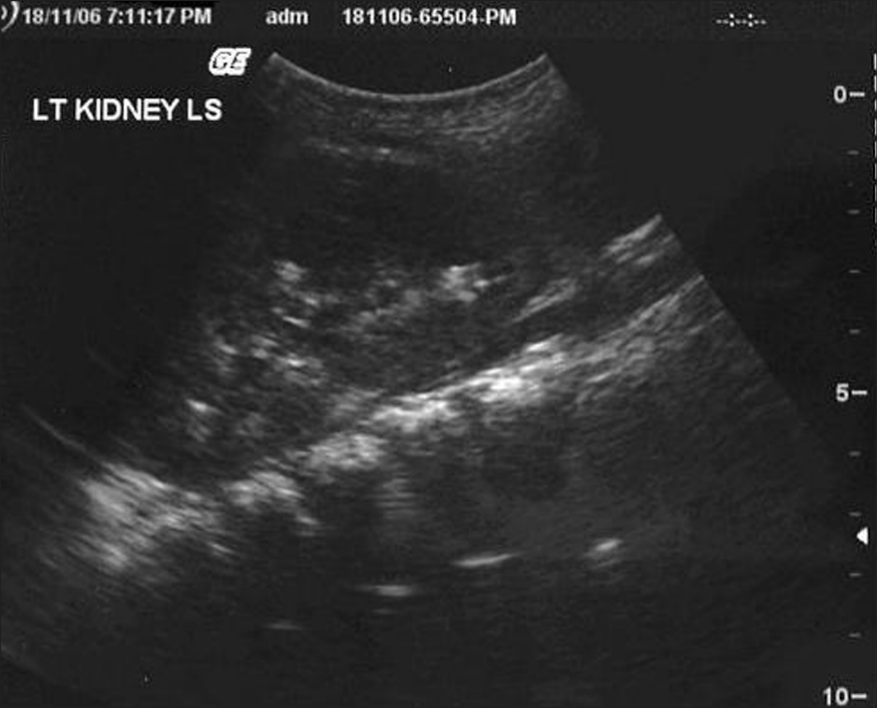
Ultrasonography of kidney showing nephrocalcinosis

Laboratory evaluation showed the following: Urine albumin-trace, sugar-nil, microscopy-no sediments; Hemoglobin 12.3 g/dl, Blood urea 21 mg/dl, serum creatinine 0.3 mg/ dl, serum sodium 137 mEq/l, serum potassium 3.4 mEq/l, serum chloride 99 mEq/l, serum bicarbonate 22 mEq/l, serum calcium 8.9 mg/dl, serum phosphate 2.0 mg/ dl (normal values for children: 4.5-6.5 mg/ dl), serum alkaline phosphatase 3966 IU/l and serum intact PTH 26.5 pg/ml (normal: 10-54). Arterial blood gas analysis showed pH 7.353, pCO_2_ 27.0 mmHg, pO_2_ 114.3 mmHg, HCO_3_ 14.6 mmol/l and simultaneous urine pH was 8.0. X-ray of wrists showed fraying and splaying of metaphyses of distal ulna and radius, old healed fractures of distal shaft of radius. Ultrasound of abdomen showed normal sized kidneys with multiple hyperechoic foci within, suggestive of calcification [Figure 2]. X-ray of abdomen showed no evidence of calcification in the region of kidneys.

A diagnosis of hypophosphatemic rickets was made and was evaluated further. The 24-h urine protein excretion was 542.8 mg/day, phosphate excretion was 437 mg/ day, β2-microglobulin excretion was 37.6 mg/ day (normal < 0.2 mg/day) and calcium excretion was 82.8 mg/day (9mg/kg/day). Examination of urine showed generalized aminoaciduria. A diagnosis of Dent's disease was made, since he met the following criteria: male gender, rickets, severe hypercalciuria (9 mg/ kg/ day), LWMP (β2-microglobulin), hypophosphatemia, phosphaturia and nephrocalcinosis.

He received potassium citrate (potassium citrate monohydrate 220 mg and citric acid monohydrate 67 mg per ml) 3 ml twice daily, calcitriol 0.25 μg on alternate days, hydrochlorothiazide 6.125 mg twice daily and sodium phosphate (Proctoclysis enema preparation; sodium dihydrogen phosphate 16%, sodium phosphate 6%, 1 ml contains approximately 50 mg of elemental phosphorus) 1 ml orally thrice daily. The dose of drugs was adjusted periodically to achieve targets. At one year, he was on sodium phosphate 1 ml five times daily, potassium citrate 4 ml thrice daily, calcitriol 0.25 μg/day and hydrochlorothiazide 6.125 mg twice daily. The details of anthropometric and biochemical measures before and after therapy are shown in Table 1. At one year, there was a marked improvement in his mobility; he was able to walk and run and the bony deformities had improved significantly. There was a gain in height and weight, serum phosphorus was near normal and serum alkaline phosphatase was reduced by 50%.

**Table 1:** Anthropometric and biochemical parameters before and after treatment

	8 mo before	0 mo	4 mo	12 mo
Weight (kg)	8.3	9	9.5	10.4
Height (cm)	70	71	75	79
S.Ca (mg/dl)	8.9	8.9	9	8.7
S.PO_4_ (mg/dl)	2.0	2.0	3.5	3.8
SAP (IU/l)	3208	3966	3070	1591
Ur Ca (mg/kg/day)	10	9	2	-

Ca = Calcium; PO_4_ = Phosphate; SAP = Serum alkaline phosphatase; Ur = Urine; mo = Months

## Discussion

The diagnosis of rickets due to Dent's disease was made in our case based on male gender, hypophosphatemia, LMWP, nephrocalcinosis and hypercalciuria. He received phosphate and calcitriol supplements. Since commercial oral phosphate preparation was not available, sodium phosphate enema preparation was administered orally which was effective and well tolerated. Hypercalciuria was abolished using hydrochlorothiazide. At 12 months, there was significant bone healing, improvement in biochemical and anthropometric parameters and improvement in mobility.

### Molecular genetics and pathogenesis

Dent's disease is caused by a mutation in renal chloride channel gene (CLCN5) located on chromosome Xp11.22.[3] In 1994, positional cloning in families with Dent's disease identified CLCN5 gene that codes for the chloride channel-5 (CLC5). To date, more than 80 distinct CLCN5 mutations have been reported.[4] However, there is no genotype-phenotype correlation, since various mutations were found to be associated with quite different clinical phenotypes. Also, there are patients with typical features of Dent's disease in whom no CLCN5 mutations could be detected, indicating a genetic heterogeneity.[4–6] CLC5 belongs to the family of voltage-gated chloride channels and has an important role in the control of membrane excitability, trans-epithelial transport, and cell volume.[4,7] CLC5 is a key mediator of chloride conductance that is necessary for early endosomal acidification and receptor-mediated reabsorption in proximal tubular cells.[7] A defect in CLC5 function results in complex array of events as described below. It results in inhibition of apical endocytosis of LMWPs; this occurs due to the slowing down of the recycling of megalin and cubulin to the brush border.[4,7] Parathyroid hormone (PTH) passes the glomerular filter and in early proximal tubule (PT) it is normally endocytosed after binding to megalin. This process is severely impaired when CLC5 is missing, resulting in an increase of luminal PTH levels in later segments of PT, resulting in enhanced stimulation of apical PTH receptors. It in turn triggers endocytosis and the lysosomal degradation of apical sodium coupled phosphate transporter NaPi-2a, resulting in phosphaturia. An augmented stimulation of apical PTH receptors stimulates the transcription of mitochondrial enzyme 1α-hydroxylase that forms the active hormone 1, 25(OH)_2_ vitamin-D_3_ from the inactive precursor 25(OH) vitamin-D_3_. Because 1, 25(OH)_2_ vitamin-D_3_ stimulates intestinal calcium absorption, an increase in its serum concentration might indirectly lead to hypercalciuria and nephrocalcinosis.[4]

### Clinical features

The clinical manifestations in Dent's disease are many, of which only LMWP is the constant feature. The prevalence of various manifestations is as follows: LMWP-100%, hypercalciuria-95%, nephrocalcinosis-74%, rickets or osteomalacia-30%, renal failure-64%, aminoaciduria-76%, glycosuria-54%, hypophosphatemia-50%, renal acidification defects-17%.[1] The disease manifests in males and female carriers are usually asymptomatic, but often show LMWP and rarely hypercalciuria. Approximately one third of affected males develop some degree of renal insufficiency, which is generally apparent by late childhood. Patients who reach end-stage renal failure do so between ages 30-40 years.[1,2] The cause of renal failure in Dent's disease remains unclear. Nephrocalcinosis and renal calculi may partly explain renal insufficiency, but other factors such as tubulopathy related to LMWP might also play a role. Approximately 25% of affected males develop rickets, which may present in infancy as seen in our case.[1] Hypophosphatemic rickets due to Dent's disease in children should be differentiated from two other conditions, namely X-linked vitamin-D resistant rickets and proximal RTA and the differentiating features between them are shown in Table 2.

**Table 2:** Differential diagnosis of hypophosphatemic rickets in children

	VDRR	Proximal RTA	Dent's disease
Gender	Both	Both	Male
Ser. phosphate	Low	Low	Low
Ser. calcium	Normal	Low	Normal
Calcitriol levels	Normal/low	Low	Normal/Mild high
Hypercalciuria	No	No	Yes
Nephrocalcinosis	No	No	Yes
Ser. PTH	Normal/mild high	High	Normal

VDRR = Vitamin-D resistant rickets; RTA = Renal tubular acidosis; PTH = Parathormone

### Therapy

The treatment of Dent's disease is complex and involves integration of therapies directed at different manifestations of the disease. The plan of therapy calls for a clear understanding of pathophysiology of the disease and simultaneous monitoring of the different aspects of therapy, since misdirected therapy can do harm. The goal of therapy is to prevent nephrocalcinosis, which could contribute to renal insufficiency and to promote bone healing. The prevention of nephrocalcinosis may be achieved by the following (i) reducing hypercalciuria, (ii) citrate supplements and (iii) correction of acidosis if present. Hydrochlorothiazide reduces hypercalciuria,[8] the dose can be titrated to achieve normal calcium excretion. Distal RTA may occur as a consequence of nephrocalcinosis and can further aggravate hypercalciuria. Bicarbonate supplement may be necessary if significant metabolic acidosis exists. Citrate is a source of bicarbonate, also has an inhibitory effect on nephrocalcinosis and is useful in preventing nephrocalcinosis. A study in the mouse model of Dent's disease showed that citrate supplements retarded the progression of renal insufficiency.[9] The healing of bone lesions is promoted by phosphate and calcitriol supplements. Elemental Phosphorus may be started at dose 20-40 mg/kg/day and should be given in evenly spaced three to five divided doses and the dose can be increased in steps to a maximum of 3500 mg/day. Calcitriol can be started at 20-40 ng/kg/day in two divided doses. Calcitriol can cause increase in gut absorption of calcium, which can aggravate hypercalciuria, which in turn may worsen nephrocalcinosis. Hence, calcitriol dose should be tightly titrated so as to avoid hypercalciuria. Phosphate supplements can increase the serum phosphate level, which in turn can decrease serum calcium by chelation. Loss of inhibitory effect of hypophosphatemia and stimulatory effect of hypocalcemia can result in an increased release of PTH. Increased PTH in blood could lead to increased PTH at the last segment of PT, which can aggravate the principal pathogenic factor in Dent's disease (see pathogenesis). The aim of therapy in Dent's disease is not to normalize serum phosphate levels, but rather to normalize serum alkaline phosphatase and achieve longitudinal growth.

## Conclusion

we report Dent's disease in a two- year- old boy, presenting with rickets. Supplements with phosphorus, calcitriol and potassium citrate resulted in remarkable improvement in mobility, growth and bony deformities. The hypercalciuria associated with Dent's disease was effectively corrected using hydrochlorothiazide.
